# Etude du strabisme chez des enfants de 0 à 15 ans suivis a Lubumbashi, République Démocratique du Congo: analyse des aspects épidémiologiques et cliniques

**DOI:** 10.11604/pamj.2015.22.66.5324

**Published:** 2015-09-23

**Authors:** Yogolelo Asani Bienvenu, Musau Nkola Angel, Mbuyi Musanzayi Sebastien, Cilundika Mulenga Philippe, Kabamba Ngombe Léon, Twite Kabange Eugene, Cham Lubamba Chami, Kalenga Muenze Kayamba Prosper, Speeg-Schatz Claude, Chenge Borasisi Gaby

**Affiliations:** 1 Université de Lubumbashi, Faculté de Médecine, Service d'Ophtalmologie, Lubumbashi, République Démocratique du Congo; 2 Université de Lubumbashi, Faculté de Médecine, Département de Santé Publique, Lubumbashi, République Démocratique du Congo; 3 Université de Lubumbashi, Faculté de Médecine, Département de Chirurgie, Lubumbashi, République Démocratique du Congo; 4 Université de Kamina, Faculté de Médecine, Département de Santé Publique, Unité de Toxicologie, Kamina, République Démocratique du Congo; 5 Université de Lubumbashi, Faculté de Médecine, Département des Sciences Biomédicales, Lubumbashi, République Démocratique du Congo; 6 Université de Strasbourg, Faculté de Médecine, Service d'Ophtalmologie, Strasbourg, France

**Keywords:** Enfant congolais, strabisme, hypermétropie, ésotropie, Congolese child, strabismus, hypermetropia, esotropia

## Abstract

**Introduction:**

Le strabisme est défini comme un syndrome à double composante: motrice et sensorielle. Le but de ce travail est de décrire les aspects épidémiologiques et cliniques du strabisme chez l'enfant congolais de 0 à 15 ans dans la ville de Lubumbashi.

**Méthodes:**

Il s'agit d'une étude descriptive longitudinale sur les aspects épidémiologiques et cliniques du strabisme chez l'enfant congolais de 0 à 15 ans dans la ville de Lubumbashi entre Décembre 2012 à Décembre 2013. Nous avons recueilli l’âge des patients, leur sexe, leur provenance, le type de strabisme, la réfraction, le fond d'oeil, les antécédents (hérédité) ainsi que le type de la déviation strabique observé sur 70 patients.

**Résultats:**

Nous avons observé 70 cas de strabisme manifeste dont 31 cas (44,28%) étaient dans la tranche d’âge comprise entre 0 et 5 ans. L’âge moyen de nos patients était de 6,7 ans avec une prédominance du sexe féminin, soit 51,42%. Le strabisme était convergent dans 65,71%, divergent dans 30%, et vertical dans 4,28%. Les ésotropies représentaient 65 cas (92,85%), quatre cas (5,71%) avaient un antécédent familial de strabisme au premier degré de parenté, 21 cas (30%) au second degré de parenté, 45 cas (64,28%) n'avaient pas cet antécédent. L'oeil gauche était le plus dominé dans 30% des cas. Les facteurs favorisant le strabisme étaient inconnus dans 54 cas (77,14%). Le strabisme était secondaire à l'hypermétropie chez 32 patients (42,71%).

**Conclusion:**

La fréquence du strabisme dans la ville de Lubumbashi chez les enfants âgés de 0 à 15 ans est de 0,50%. Comme dans la plupart des études sur le strabisme de l'enfant, c'est l’ésotropie qui est la déviation la plus commune.

## Introduction

Il y a strabisme lorsque les axes visuels des deux yeux ne sont pas orientés vers le même objet de fixation. De ce fait deux phénomènes sont donc à la base: le phénomène moteur (perte de parallélisme) et le phénomène sensoriel (correspondance rétinienne anormale). Le strabisme est évidemment plus fréquent dans l'enfance mais ce qu'il faut souligner, ce sont les enjeux qui sont en causes. En premier lieu, il faut se rappeler qu'un strabisme peut révéler une pathologie potentiellement grave. L'amblyopie (diminution de l'acuité visuelle non améliorable par les verres correcteurs) induite par toute anomalie organique peut entrainer une déviation de l'oeil. Il est anormal pour un nourrisson de loucher, et un avis spécialisé est toujours nécessaire pour réaliser un examen des structures oculaires après dilatation de la pupille à la recherche d'une pathologie organique qui peut aller de la cataracte congénitale au rétinoblastome sans oublier de rechercher un défaut réfractif. L'enjeu de strabisme est dans la majorité des cas fonctionnel: la prise en charge thérapeutique spécialisé de toute déviation d'un oeil, par traitement optique, orthoptique, chirurgical le cas échéant, a pour but premier le maintient ou la restauration d'un état sensoriel satisfaisant [1]. On estime en effet que 4% des enfants souffriraient de strabisme. C'est un problème de santé publique pour lequel tous lesacteurs doivent être mobilisés [2]. La fréquence du strabisme est comprise entre 0,99 et 2,6% en Europe. Matsuo, l'a estimé à 1,26% en 2003 et 0,99% en 2005 [3]. Dans notre milieu, aucun travail n'a été fait sur le strabisme chez l'enfant. Notre étude se propose donc de relever les aspects épidémiologiques et cliniques du strabisme chez l'enfant de 0 à 15 ans dans la ville de Lubumbashi.

## Méthodes

Cette étude a été menée à Lubumbashi (R.D.Congo) au centre ophtalmologique des cliniques universitaires de Lubumbashi. Il s'agit d'une étude descriptive longitudinale qui s'est déroulé de Décembre 2012 à Décembre 2013 soit une période de 12 mois. Notre population d’étude était constituée des enfants congolais âgés de 0 à 15 ans, soit 102 enfants dont 32 perdus de vue sur un total de 20579 enfants. Comme critères d'inclusion, nous avons retenu la présence d'un strabisme manifeste chez les enfants congolais âgés de 0 à 15ans venus en consultation pendant la période de recensement et dont les parents ont adhéré aux questionnaires. Pour récolter les données, nous avons fait recours à une fiche de récolte comprenant les données sociodémographiques de nos enfants telles que l’âge, le sexe, la provenance, les antécédents de strabisme dans la famille restreinte ou élargie ainsi que les données de l'examen objectif telles que la réfraction sous collyre atropine (<3 ans: 0,3%, entre 3 et 5 ans: 0,5%, >5 ans 1%: 1goutte 2x par jour pendant une semaine), le fond d'oeil, la déviation oculomotrice. L'analyse des résultats a été faite à l'aide du logiciel Excel 2007 et épi-info version 7.0.8.3. Les résultats ont été présentés sous forme de tableaux.

## Résultats

Le strabisme manifeste a été retrouvé chez 102enfants sur un effectif de 20579 enfants; soit 0,5% (Tableau 1). En rapport avec l’âge des patients, la plupart des strabiques sont compris dans la tranche d’âge de 0-5 ans soit 44,28% des cas (Tableau 2). L’âge moyen était de 6,7 ans avec un écart-type de 4,5 ans. La médiane était de 7 ans et le mode de 4 ans. Le sex-ratio était de 1,06 (un homme pour 1,06 femme); le type de strabisme observé était l’ésotropie. Tenant compte de degré de parenté (Tableau 3), la transmission du premier degré de parenté était observée dans les deux sexes dans des proportions égales, soit 2,85%; par contre la transmission du deuxième degré de parenté était observée dans le sexe féminin, soit 17,14%.

**Tableau 1:** Répartition des enfants selon la présence du strabisme

Strabisme	Effectif	Pourcentage
Présent	102	0,5
Absent	20509	99,66
Total	20579	100

**Tableau 2:** Répartition des patients strabiques selon les différentes tranches d’âge et le type du strabisme

Age (an)	Esotropie	Exotropie	Vertical	Total
0-5	22(31,42%)	8(11,42%)	1(1,42%)	31(44,28%)
6-10	13(18,57%)	7(10%)	2(2,85%)	22(31,42%)
11-15	11(15,17%)	6(8,57%)	0(0,00%)	17(24,28%)
**Total**	**46(65,71%)**	**21(30%)**	**3(4,28%)**	**70(100%)**

**Tableau 3:** Répartition des patients strabiques selon le sexe et le degré de transmission du strabisme

	Masculin	Féminin
1° degré de parenté	2(2,85%)	2(2,85%)
2° degré de parenté	9(12,85%)	12(17,14%)
**Aucune**	**34(48,57%)**	**36(51,42%)**

Les deux sexes étaient observés dans les mêmes proportions pour le strabisme précoce soit 37,14% des cas. Le strabisme tardif était observé dans le sexe féminin, soit 14,28% (p = 0,171) (Tableau 4). A l'examen du type de strabisme selon le sexe (Tableau 5), l’ésotropie était rencontrée dans le sexe masculin, soit 34,28% par contre l'exotropie et le strabisme vertical étaientrencontrés dans le sexe féminin, respectivement 15,71% et 4,28% (p= 0,120). En examinant les vices de réfraction selon le sexe (Tableau 5), l'hypermétropie a été observée dans 24,28% chez les garçons et 21,42% chez les filles (p = 0,6199). L'astigmatisme myopique a été, quant à lui retrouvé dans 3 cas chez les filles (4,28%) et 2 cas chez les garçons (2,85%). Les perdus de vue ont été signalé dans 24,22% chez les filles et 21,42% chez les garçons (Tableau 6).

**Tableau 4:** Répartition des patients strabiques selon le sexe et l’âge d'apparition du strabisme

	Masculin	Féminin	Total
Strabisme précoce (0 à 8mois)	26(37,14%)	26(37,14%)	52(74,28%)
Strabisme tardif (après 2ans ½ et à 3ans)	8(11,42%)	10(14,28%)	18(25,71%)
Total	**34(48,57%)**	**36(51,42%)**	**70(100%)**

**Tableau 5:** Répartition des patients strabiques selon le sexe et le type de strabisme

	Masculin	Féminin	Total
Esotropie	24(34,28%)	22(31,42%)	46(65,71%)
Exotropie	10(14,28%)	11(15,17%)	21(30%)
Vertical	0(0,00%)	3(4,28%)	3(4,28%)
**Total**	**34(48,57%)**	**36(51,42%)**	**70(100%)**

**Tableau 6:** Répartition des patients strabiques selon le sexe et les vices de réfraction

	Masculin	Féminin	Total
Hypermétropie	17(24,28%)	15(21,42%)	32(42,71%)
Myopie	0(0,00%)	1(1,42%)	1(1,42%)
Astigmatisme myopique	2(2,85%)	3(4,28%)	5(7,14%)
Perdus de vue	15(21,42%)	17(24,22%)	32(42,71%)
**Total**	**34(48,57%)**	**36(51,42%)**	**70(100%)**

## Discussion

Le Tableau 1 a montré que la fréquence du strabisme est de 0,5%. Ce chiffre rejoint celui trouvé par Wedner [4] en Tanzanie. Mais d'autres auteurs ont eu des fréquences une ou trois fois supérieure à la notre; il s'agit de Chia [5] en Chine, Lithander [6] dans le Sultanat d'Oman et Garvey [7] aux USA respectivement de 0,80%, 0,90% et 1,5%. Cette différence de fréquence peut être attribuée à la différence de taille de l’échantillon ou des critères d'inclusion.

L'analyse du Tableau 2 montre que 31,42% des patients strabiques étaient ésotropes dans la tranche d’âge comprise entre 0 et 5 ans. Ceci pourrait être lié par le fait que les muscles adducteurs de l'oeil sont beaucoup plus fonctionnels dans cette tranche d’âge. L’âge moyen dans notre étude était de 6,7 ans avec une prédominance féminine; cela se rapproche de l’étude d'Azonobi [8] au Nigéria qui a trouvé un âge moyen de 6 ans mais avec une prédominance masculine, et de celui de Faghili [9] en Iran qui a trouvé une prédominance féminine avec un âge moyen de 13,2 ans.

D'après le Tableau 3, la transmission du deuxième degré de parenté était observée, soit 17,14%. Ces résultats se rapprochent de ceux de bien d'autres auteurs notamment Mvogo [10] au Caméroun qui a trouvé 22,78%, Regoda et Sefic (2012) [11] en Bosnie-Herzegovine a signalé quant à lui 68,14%. La littérature indique qu'il existe une composante héréditaire dans l’étiopathogénie du strabisme, mais ses sites génétiques n'ont pas encore été identifiés. Il y a une forte espérance en ce sens qu'avec le décodage du génome humain et les progrès de la biologie moléculaire, on arrive à identifier les gènes responsable du strabisme. Cependant, des études épidémiologiques et cliniques peuvent permettre de suspecter une origine génétique du strabisme. L’étude des cas familiaux est importante car elle permet à des groupes à haut risque d’être dépistés. Elle permet ainsi de lutter efficacement contre l'amblyopie grâce à une détection et un traitement précoce. Une transmission multifactorielle est pour le moment le plus probable: trois locus de susceptibilité (7p22.1, 4q28.3, 7q31.2) ont été déterminés sans qu'un gène n'ait été isolé. En attendant, l'on se contente de rechercher les facteurs de risque dans les familles de patients strabiques et organiser la prise en charge de la maladie Péchereau [12].

Le Tableau 4 montre que le strabisme précoce a été retrouvé dans 74,28% des cas. Cette fréquence élevée est rapportée à des degrés variables par différents auteurs. Merino [13] en Espagne a trouvé 63,63% de strabisme précoce. Greenberg [14] aux USA a signalé dans son étude que le strabisme précoce était de 8,1 sur les 385 enfants examinés (31/385). Thouvenin [15] en France a rapporté 34% de strabisme précoce de 622 cas des désordres oculomoteurs. Les hypothèses sont nombreuses et discutées, mais plusieurs auteurs s'accordent et pensent qu'il s'agit d'un trouble de la maturation des voies visuelles et que le tableau clinique caractéristique est dû à la précocité d'installation du strabisme dans la période de développement de la vision binoculaire.

En analysant le Tableau 5, l’ésotropie (67,71%) figure parmi le type le plus fréquent de strabismesuivi de l'exotropie (30%) et enfin le strabisme vertical (4,28%). Kac [16] a trouvé que l’ésotropie était le désalignement le plus fréquent (44,52%) dans une étude rétrospective de 935 dossiers de patients examinés au secteur de strabisme en 2005 de l'hôpital Servidor Publica au Brésil. Pour sa part, Yan Ke Bao [17] a trouvé que 32 cas soient 66,67% d'exotropes et 16 cas soient 33,33% d’ésotropes et 15 cas soient 31,25% avaient souffert de deux strabismes dans une enquête faite sur les élèves Kazzak en Chine, sur un total de 4125 enfants âgés de 4 à 14 ans. Regoda et Sefic [11] rapporte également que c'est l’ésotropie qui est le type le plus fréquent de strabisme en observant à la clinique externe « Profi Optique » les cas de strabismes chez les enfants. Nos résultats et ceux des autres auteurs des pays européens trouvent l’ésotropie comme le désalignement le plus fréquent chez l'enfant tout simplement grâce à la synergie accommodation-convergence, phénomène qui est présent que dans la vision de près et mettant ainsi les muscles adducteurs de l'oeil en jeu d'où convergence (ésotropie).

Le Tableau 6 a montré que l'hypermétropie était le vice de réfraction le plus observé, soit 24,28% chez les garçons et 21,42% chez les filles. Nos résultats sont comparables à ceux rapportés par d'autres auteurs comme Mvogo [10] dans une étude de l'exotropie chez le noir camerounais où l'hypermétropie était la forme commune des amétropies (59,86%). Pour sa part, Sandfeld Nielsen [18] au Danemark a démontré que l'astigmatisme représentait 20,6% de vice de réfraction suivi de l'hypermétropie 15,3% et enfin la myopie 10,8% dans une étude sur les aspects des erreurs de réfraction et strabisme réalisée chez des enfants avec retard de développement. Nos résultats sont similaires à ceux d'autres auteurs dans le sens que c'est l'hypermétropie qui est le vice de réfraction le plus observé chez l'enfant strabique. En effet, le strabisme convergent est plus fréquent chez l'enfant (90%), il est secondaire à l'hypermétropie qui est spontanément corrigée par l'accommodation cristallinienne suite à la mise en jeu de la synergie accommodation-convergence. Mais en présence de forte hypermétropie (>2,50 dioptries), l'enfant va recourir à un excès d'accommodation pour voir net, ce qui entraine une convergence accommodative excessive en permanence et qui va devenir à la longue définitive, d'où strabisme. Cependant, Donders lui-même ne faisait pas de l'hypermétropie le facteur exclusif de la déviation strabique, mais un facteur déclenchant sur terrain prédisposé, où la fusion n’était pas assez forte pour maintenir le parallélisme des axes visuels [19].

## Conclusion

Au terme de notre travail, nous retenons que le strabisme chez l'enfant congolais âgé de 0 à 15 ans dans la ville de Lubumbashi représente 0,50% de fréquence. L’âge moyen de notre population d’étude était de 6,7 ans. La plupart des strabiques étaient retrouvés dans la tranche d’âge de 0 à 5 ans. Le strabisme était observé dans le sexe féminin avec 51,42% et dans le sexe masculin avec 48,57%. L’ésotropie était le type de strabisme le plus observé avec 65,71% dans l'ensemble; 34,28% dans le sexe féminin et 31,42% dans le sexe masculin. Le strabisme précoce représente 74,28% dans l'ensemble avec 37,14% dans les deux sexes. La transmission du strabisme était observée dans le deuxième degré de parenté avec 29,99% environ 30%.
